# Ultraviolet Radiation and Chronic Inflammation—Molecules and Mechanisms Involved in Skin Carcinogenesis: A Narrative Review

**DOI:** 10.3390/life11040326

**Published:** 2021-04-08

**Authors:** Magdalena Ciążyńska, Irmina Olejniczak-Staruch, Dorota Sobolewska-Sztychny, Joanna Narbutt, Małgorzata Skibińska, Aleksandra Lesiak

**Affiliations:** 1 Department of Proliferative Diseases, Nicolaus Copernicus Multidisciplinary Centre for Oncology and Traumatology, 93-513 Łódź, Poland; 2 Department of Dermatology, Pediatric Dermatology and Dermatological Oncology, Medical University of Łódź, 90-419 Łódź, Poland; owczarko.witko@wp.pl (I.O.-S.); wrobelo.katarzyna@wp.pl (D.S.-S.); joanka.narbu@wp.pl (J.N.); skibka.malgo@wp.pl (M.S.); leska.leska.ola@wp.pl (A.L.)

**Keywords:** chronic inflammation, inflammasome, skin carcinogenesis, melanoma, non-melanoma skin cancer

## Abstract

The process of skin carcinogenesis is still not fully understood. Both experimental and epidemiological evidence indicate that chronic inflammation is one of the hallmarks of microenvironmental-agent-mediated skin cancers and contributes to its development. Maintaining an inflammatory microenvironment is a condition leading to tumor formation. Multiple studies focus on the molecular pathways activating tumorigenesis by inflammation and indicate several biomarkers and factors that can improve diagnostic and prognostic processes in oncology and dermatology. Reactive oxygen species produced by ultraviolet radiation, oxidizers, or metabolic processes can damage cells and initiate pro-inflammatory cascades. Considering the potential role of inflammation in cancer development and metastasis, the identification of early mechanisms involved in carcinogenesis is crucial for clinical practice and scientific research. Moreover, it could lead to the progress of advanced skin cancer therapies. We focus on a comprehensive analysis of available evidence and on understanding how chronic inflammation and ultraviolet radiation can result in skin carcinogenesis. We present the inflammatory environment as complex molecular networks triggering tumorigenesis and constituting therapeutic targets.

## 1. Introduction

The skin is the largest organ of the body with a strategic location, being a barrier between internal tissues and an external environment. The skin maintains an organism’s homeostasis and ultimately the organism’s survival. It protects the organism from radiation, protects the body from mechanical stress, and forms the main wall against pathogens [1]. Undoubtedly, the disturbances of skin may induce inflammation, which is described by the infiltration of leukocytes and plasma into tissue undergoing disrupted homeostasis [2]. However, acute inflammation responds to the induced changes by removing the cause of the impairment and restoring homeostasis to the affected tissue. Chronic inflammation may promote malignant cells’ transformation and can further initiate tumor formation [3,4]. Ultraviolet radiation (UVR) from sunlight causes the most harmful effects on human health [5]. The link between URV and skin carcinogenesis has been presented within multiple epidemiological research studies. UV light influences the homeostasis of cells and tissue because of its damaging effect on DNA integrity, its modification of the expression of many genes, and its generation of reactive oxygen species (ROS). The DNA repair system protects cells from damage caused by UVR. Under normal conditions, ROS mediate crucial valuable responses, such as destroying microorganisms [6] and managing cellular growth, differentiation, and migration, which regulate cellular adhesion and vascular tone. When their production is prolonged or elevated, ROS stimulate tissue inflammation and induce intracellular protein complexes, such as inflammasomes activation [7]. Excessive levels of ROS result in oxidative stress [6,8], which is a disproportion between the ROS’ systemic manifestation and the organism’s capability to detoxify the reactive intermediates and to repair the consequential damage.

Activated keratinocytes with resident dendritic cells (DCs), melanocytes, and Langerhans cells (LCs) produce pro-inflammatory cytokines that are involved in the upregulation of inflammatory reactions and that modulate adaptive and innate immune responses [9,10]. All the immune related bioactive molecules such as cytokines, growth factors, and chemokines infiltrating the tumor microenvironment have an autocrine as well as a paracrine effect that deregulates a local milieu, initiating uncontrolled inflammation and therefore favoring the tumorigenesis process. Various lymphoid and myeloid cells penetrate the tumor microenvironment and have contradictory effects on tumor progression [11,12]. Chronic inflammation is governed by regulatory T cells, T helper (Th)2 cells that secrete tumorigenic factors including transforming growth factor beta (TGF)-β and interleukin-4 (IL-4), IL-6, IL-10, and IL-13, while acute inflammation is connected with Th1-polarized T lymphocytes activated by innate immune cells, producing mostly antitumor molecules such as interferon (IFN) γ or IL 2. Inflammatory mediators, such as tumor necrosis factor (TNF)-α, IL-6, IL-10, and TGF-β, participate in tumor initiation and progression [13]. Chronic inflammation maintains the pro-tumoral environment [14,15]. Skin that interacts with various environmental factors and is exposed to chronic inflammation triggers various processes underlying tumorigenesis. A specific inflammatory microenvironment within skin cancer also inhibits natural antitumor immunity and reduces the effectiveness of therapy.

Cutaneous keratinocyte cancers including basal cell carcinoma (BCC) and squamous cell carcinoma (SCC) of the head and neck currently represent the most common form of neoplasm in Caucasians. The rapid increase is based on UVR exposure, accounting for almost 90% of non-melanoma skin cancer cases. BCC is more connected with intermittent sun exposure in childhood, accounting in some populations for up to 80% of all carcinomas, while SCC development is more related to exposure to chronic UVR [16,17,18]. Taking into consideration that skin cancer is one of the most common malignancies, whose incidence is rapidly increasing worldwide, analysis of inflammatory background of cutaneous keratinocyte cancers and melanomas and their complex molecular networks could act as triggers of tumorigenesis or therapeutic targets. In this review, we aim to review and conclude the current data of inflammatory signaling and its role in skin carcinogenesis, with a detailed analysis of the role of inflammation in the development of different skin cancers.

## 2. Ultraviolet Radiation and Skin Components Involved in Inflammation

All living organisms on our planet are exposed to solar radiation, which includes ultraviolet radiation. UVR can affect the human system both positively and negatively. The advantages of exposure to UVR are primarily the synthesis of vitamin D [19,20]. The harmful properties of ultraviolet radiation include mainly the effects of this factor on the skin in the form of sunburn, photodermatosis, photoaging, and the formation of precancerous and neoplastic skin lesions. To respectively discuss the relationship between chronic inflammation induced by UVR and skin carcinogenesis, it is first crucial to present the functions of the skin and details about its role and structure, which determine UVR characteristics.

UVR is one of the strongest causal risk factors for skin cancer development. Nitrogen and oxygen molecules present in the earth’s atmosphere absorb UVC radiation almost completely and 90% of the UVB radiation, which means that only a small part of the UVR reaching the earth’s surface is composed predominantly of 95% UVA radiation, with only 5% UVB radiation [21]. UVA radiation causes erythema very quickly and is irritating to the conjunctiva and the cornea of the eye. UVA and UVB can cause DNA damage and inflammation that over time may lead to skin cancer development [22,23]. Defective DNA is usually restored by a nucleotide excision repair pathway, while a faulty repair mechanism for broken DNA may lead to cancer development [24]. UV radiation acts as a connection between inflammation and skin cancer, as its exposure affects immunological functions in skin [25]. The data in the available literature in most cases do not distinguish between UVA and UVB radiation [26]. However, recent studies have shown inconsistent and varying results, some of which have found that UVB radiation is much more cytotoxic and mutagenic to skin tissue [27,28]. It is recognized that UVA radiation is over 1000 times less mutagenic than UVB radiation [29]. It penetrates deeply into the skin from all ranges of UV radiation and increases the harmful effects of UVB emission. Additionally, it affects fibroblasts, dendritic cells of the skin, vascular endothelial cells, T-lymphocytes, mast cells, and granulocytes [30]. UVA causes photoallergic and phototoxic reactions. It contributes to the formation of free radicals, and due to its destructive effect on DNA, it also has mutagenic and carcinogenic properties [31]. More than 50% of UVA radiation penetrates the reticulate and papillary layers of the skin, while 90% of UVB radiation is retained by the horny layer of the epidermis. UVB radiation acts on the superficial layers of the skin down to the level of the basal layer. By affecting mainly keratinocytes, melanocytes, and Langerhans cells, it causes burns and skin erythema and contributes to DNA structure damage.

UVA radiation leads to the destruction of the genetic material of the cell, under the influence of the produced ROS. As a result of the action of these forms, oxidative products are formed that have mutagenic properties, and as a result, the process of carcinogenesis within epidermal cells is initiated [31,32]. The cytotoxic effect of exposure to UVA radiation is less marked than UVB radiation since DNA is not a UVA chromophore [33] and its genotoxic effect is mediated through an indirect mechanism. The production of ROS in skin exposed to UVA causes oxidative stress in keratinocytes, which results in irreversible damage to keratinocyte stem cells, which are then transferred to “daughter cells” [34].

The destructive effect of UVB radiation results from the fact that the DNA covering the aromatic rings is a chromophore absorbing UVB radiation, which results in photoproducts such as the 6,4-pyrimidone (6,4-PP) photoproduct and cyclobutanol pyrimidine dimers (CPDs) [35,36]. What is more, the destructive effect of UVB radiation consists of breaking the bonds between the pyrimidine bases, resulting in the formation of cyclobutane pyrimidine or dipyrimidine dimers, which show mutagenic properties by disrupting the elongation of transcription processes. Failure to repair damaged DNA contributes to the formation of permanent mutations. In addition, like UVA radiation, exposure to UVB radiation may generate ROS that could damage DNA and protein molecules as well as lipids [37,38,39,40]. ROS cause oxidative stress, inducing epidermal inflammation, which results in the development of skin cancer [39,40]. Importantly, under the influence of UVB rays, ROS are formed at a much lower rate in comparison with UVA radiation [39,41].

The role of the aryl hydrocarbon receptor (AHR) for filaggrin production and its contribution to the skin barrier has already been discovered [42,43]. Moreover, the UV damage response by keratinocytes has been reported as well [44]. AHR signaling is critical for healthy skin, as it is involved in immunity and DNA damage response [45]. It is also involved in skin pathogenesis, especially in overshooting inflammation, UV skin damage, and the induction of Treg cells by UVR and skin cancer. AHR-mediated resistance to UVB radiation-induced apoptosis could be most related to skin photocarcinogenesis [46]. AHR can desensitize keratinocytes to UVB-induced apoptosis signaling [47]. Furthermore, it was revealed that the AHR suppresses pyrimidine dimer repair in vitro and in vivo and blocks the formation of double strand breaks that lead to apoptosis [45]. Remarkably, AHR knockout mice exhibited 50% fewer UVB-induced cutaneous squamous cell carcinomas than wild-type mice. Thus, AHR impacts DNA damage-dependent responses in UVB-irradiated keratinocyte carcinoma and critically contributes to skin photocarcinogenesis in mice [45].

Various external factors, mainly UVR, may activate keratinocytes, the main cellular components of the skin, and other components minor in number, such as melanocytes and skin resident LCs and DCs. Afterward, cell stimulation secretes immune-related molecules such as growth factors, cytokines, and chemokines, which alter the local microenvironment, predisposing one to inflammation and subsequent tumorigenesis [48]. Stimulated keratinocytes may play two roles in activating T cells. On the one hand, the upregulation of T cell functions is connected with the secretion of granulocyte-macrophage colony stimulating factor (GM-CSF), IL-1, IL-6, IL-7, IL-12, IL-15, IL-18, and TNF-α by these skin cells. In turn, bioactive molecules such as IL-1Rα, IL-10, chemokine (CXC motif) ligand 10 (CXCL10), α-melanocytes stimulating hormone (α-MSH), prostaglandin E2 (PGE2), and anti-IL-1 secreted by keratinocytes can lower T cell functions [49].

In addition to keratinocytes, recent studies have indicated that human sebocytes are not only passive during skin inflammation, which was previously believed, but are active modulators of immune system cell responses via cytokine and chemokine secretion [50,51,52]. The main role of sebocytes is the production and secretion of lipids that moisturize skin. Nevertheless, it was stated recently that CD4+ IL-17+ T cells are connected in acne lesions with sebocytes and secrete chemokines, such as CXCL8, which interact with neutrophils, T lymphocytes, and monocytes. Furthermore, other cytokines such as IL-1β, TGF-β, and IL-6, also secreted by sebocytes, activate the differentiation of CD4+ CD45RA+ naive T cells to Th17 cells without disturbing memory T cells. For the first time, a direct correlation between the sebaceous cells of the skin and the activation of chronic inflammatory processes has been demonstrated, leading to a pro-carcinogenic process [50].

Fibroblasts are another skin cell population connecting tumorigenesis and inflammation in cutaneous tissue. These cells are involved in wound healing by producing and depositing collagen, which causes fibrosis. Fibrous connective tissue is the microenvironment of pro-carcinogenesis [53].

Recent studies have shown that gender plays an important role in inflammation and predisposition to skin cancer development. Qing-Yuan et al. [54] revealed that both ROS production and IL-6 and TNF-α protein expressions in skin exposed to UVR were more elevated in male mouse models compared with females. Sex hormones could be modulators of skin carcinogenesis, as there are gender differences in tumor development in skin exposed to UVR. It was observed that estrogen could play a protective role in skin physiology [55]. The anti-inflammatory activity of estrogen was attributed to the interference of nuclear factor kappa B (NF-κB) transcriptional activity [56,57].

It is worth emphasizing that exposure to UVB radiation causes immunologic deviations that inhibit the host immune system from recognizing the tumor and lead to immunologic tolerance. UVR-induced immune suppression is a crucial risk factor for the development of skin cancer. It has been confirmed in multiple studies that UVR-induced by tolerance involves the appearance of regulatory T cells within the tumor-bearing hosts, which inhibit immunologic recognition of the tumor. UVR stimulates keratinocytes to release immunosuppressive soluble mediators, including IL-10. These mediators may then enter the circulation and may suppress the immune system in a systemic manner. The immunosuppressive effects of IL-10 could be factors by which these tumors escape immunologic control [58]. Antigen-presenting cells (APCs) are prevented from performing their normal function by cytokines, most notably IL-10 and TNF-α, released by keratinocytes and mast cells.

UV-induced DNA damage, predominantly in the form of CPDs, has been recognized as a crucial molecular trigger for the suppression of immune responses and the initiation of UV-induced carcinogenesis [59]. Moreover, it is a significant event in the migration of antigen-presenting cells (such as Langerhans cells in the epidermis) from the skin to the draining lymph nodes. DNA damage in antigen-presenting cells impairs their capacity to present antigen, which in turn results in a lack of sensitization [60]. CPD-containing antigen-presenting cells were observed in the draining lymph nodes of UV-exposed mice [61]. These antigen-presenting cells were determined to be of epidermal origin and to exhibit an impaired ability to present antigen. Thus, UV-induced DNA damage is one of the earliest molecular events in the development of immune suppression.

The immunoregulatory cytokine IL-12 may remove or repair UV-induced DNA damage in the skin [62]. Depending on the severity of the DNA damage following UV exposure of the skin, keratinocytes in the skin can progress to either apoptosis or DNA repair pathways. If the DNA damage is irreparable, the cell cycle is arrested and the keratinocyte is transformed into a sunburn cell, which is an early morphologic indicator of epidermal cell apoptosis. It was revealed that the endogenous DNA repair mechanism requires the presence of IL-12 in mice, which supports the concept that IL-12 could repair UVB-induced DNA damage [62].

## 3. Signaling Pathways Connected with Skin Cancer and Inflammation

Inflammation is involved in almost all phases of cancer development, including initiation, malignant conversion, promotion, tissue invasion, progression, and metastasis [63]. Inflammatory responses are responsible for almost 20% of the global cancer burden worldwide [14,64,65]. Thus, tumor-related inflammation represents one of the seven hallmarks of cancer that are required for tumor development [66]. Biochemical studies and genetic knockout mice models have indicated that two transcription factors, signal transducer and activator of transcription 3 (STAT3) and NF-κB, are the major factors associated with inflammation related to cancer [63]. The tumor inflammatory microenvironment plays an important role in carcinogenesis by enhancing the proliferation of mutated cells. Inflammatory cells can enhance mutation rates by inducing DNA damage and genomic instability through ROS and reactive nitrogen intermediates [63]. Hypoxic conditions in a cancer microenvironment stimulate the angiogenesis required for tumor growth by inducing the expression of hypoxia-inducible factor-1 alpha (HIF-1α) [63].

Inflammatory signaling pathways connected with tumors consist of an intrinsic and extrinsic part. As presented in the previous part of the review, chronic inflammation leading to malignancy is induced by the production of oxidative stress in response to radiation such as UVR, mechanical and chemical factors, or pathogens. These factor-promoting conditions, which increase the risk of cancer, constitute the extrinsic pathway. Skin exposure to various provocative agents induces the infiltration of neutrophils, which are key producers of reactive nitrogen species and ROS involved in all phases of carcinogenesis. Elevated levels of ROS can activate nuclear factor kappa B (NF-κB), phosphoinositide 3-kinase/Akt8 virus oncogene cellular homolog (PI3K/Akt) pathways, activator protein-1 (AP-1), and extracellular signal-regulated kinase-/mitogen-activated protein kinase (ERK/MAPK).

The intrinsic pathway is connected with genetic changes that predispose one to neoplasia, such as mutation activation in oncogenes (BRAF, N-ras, H-ras, and c-MYC), the inactivation of tumor suppressor genes, or chromosomal amplification or rearrangement. As a result of these genetic transformations, there is an increased production of pro-inflammatory cytokines, thereby producing an inflammatory environment in cancers.

The activation of these two pathways results in the stimulation of STAT3, transcription factors, mainly NF-κB, and signal transducer in cancer cells. NF-κB and STAT3 regulate the inflammation, survival, proliferation, invasion, angiogenesis, and metastatic potential of cancers cells. Therefore, understanding the molecular mechanism of STAT3 and NF-κB linked with tumors can enable proposals for new chemotherapeutic as well as chemo-preventive approaches. The constitutive activities of NF-κB have been confirmed in many human tumors [67]. Cutaneous SCC cells are stated to constitutively express activated NF-κB [68].

**Figure 1:**
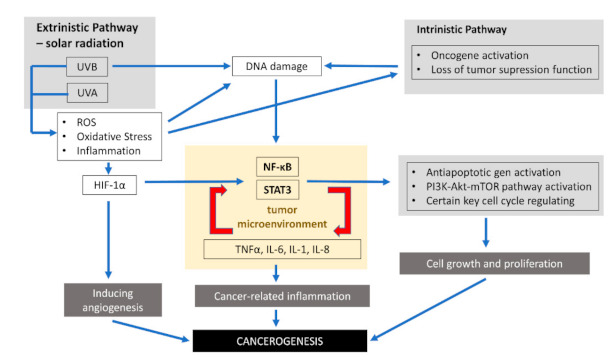
Signaling inflammation pathways inducted by ultraviolet radiation (UVR) associated with skin cancer development.

NF-κB signaling promotes cancer development in two ways: first through its activity in cancer cells and second through its effect on the immune cells [69]. NF-κB activation upregulates main inflammatory factors, including IL-1, IL-6, IL-8, and TNF-α. Inflammatory factors, on the other hand, are also activators of NF-κB. The activation of NF-κB in immune cells leads to the expression and then creation of several pro-inflammatory cytokines [69,70,71,72,73] and the upregulation of the c-FLIP. Moreover, NF-κB boosts skin tumor cell survival and confers its resistance to RAF inhibitor treatment [72,74]. Furthermore, the activation of NF-κB in tumor cells increases their survival due to the increased activity of anti-apoptotic genes including c-FLIP, c-IAP2, Bcl-2, Bcl-xL, and A1 [69,75,76]. In the skin, there are many genes involved in the initiation of inflammation, the activation of which depends on NF-κB, e.g., genes for vascular cell adhesion molecule-1 (VCAM-1), intercellular adhesion molecule-1 (ICAM-1), E-selectin, and urokinase plasminogen activator (uPA) [77]. Recent data indicate that the function supporting the survival of NF-κB observed in tumors is associated with its functional interaction with the PI3K-AKT-mTOR signaling pathway, which is the crucial element in promoting cell proliferation and cell growth. Additionally, certain key cell cycle regulating genes such as cyclin D1, D2, D3, E1, c-myc, CDK2, CDK4, and CDK6 are also controlled by NF-κB [78]. Thus, NF-κB is closely connected to the whole process of carcinogenesis [79,80]. Signaling inflammation pathways inducted by UVR associated with the development of skin cancer are presented in Figure 1. Table 1 presents cytokines involved in different processes of carcinogenesis.

The STAT family of proteins has a well-established function in inducing and maintaining the pro-carcinogenic inflammatory microenvironment. IL-6 signaling induces and activates STAT3, supporting cancer cell survival and proliferation by upregulating expression of the anti-apoptotic protein, Bcl-2, in various tumor cell lines, such as melanoma cells. Disruption of STAT3 signaling has been shown to block the transformation of fibroblasts by the SRC oncoprotein [81,82]. This discovery confirmed the key role of the STAT3 protein in the development of oncogenesis. Noteworthy, activating mutations were not found in the genes encoding NF-κB and STAT3 in solid tumors. Disturbances in their activity are caused by mutation that occurs either in genes encoding negative regulators or in upstream mediators.

## 4. Myeloid and Lymphoid Cells Involved in Inflammation

Myeloid and lymphoid cells infiltrating the tumor stroma include dendritic cells (DCs), natural killer cells (NK cells), Th1, Th17, CTL, and Treg lymphocytes, monocytes, and macrophages [83]. These cells are the main source of cytokine production, and they support or limit carcinogenesis. Among the cytokines that promote the emergence and development of tumors caused by inflammation and the activation of the survival and proliferation mechanisms in neoplastic cells, we can distinguish TNFα, IL-1, IL-6, IL-17, and IL-23, which are secreted by the monocytes, macrophages, and Th17 lymphocytes infiltrating tumors [11,12,14,84]. Among the cells infiltrating the tumor stroma, there are also immune cells such as Th1 lymphocytes and NK cells that recognize tumor-specific surface patterns and eliminate tumor cells [85,86,87]. IFN-γ and IL-12 prevent the development of skin cancer.

## 5. Inflammatory Molecules Involved in Skin Carcinogenesis

Available data indicate that inflammatory molecules are involved in carcinogenesis, while some products created during inflammation can modify or even damage DNA, lipids, and proteins [65]. Inflammations are involved in providing proliferative and survival signals to activate initiated cells, leading to cancer development [68]. In addition to the previously mentioned crucial role of the NF-κB protein in inflammation, other molecular inflammatory molecules such as cytokines, chemokines, inflammatory enzymes (cyclooxygenase (COX)-2, matrix metalloproteinases), anti-apoptotic proteins, oncogenes, and transcription factors (CREB, AP-1, STAT3) are involved in the regulation of tumor cell proliferation, transformation, and survival [88].

RAS genes, which are mutated in 25% of all malignancies and which have mediated tumor formations, are usually connected to the upregulation of chemokines and cytokines, initiating an inflammatory response related to tumorigenesis [89]. RAS genes contribute to the promotion of tumor growth in malignant cells and cell proliferation. Throughout inflammatory stimuli, RAS genes induce the expression of several inflammatory gene products, such as pro-inflammatory cytokines (IL-1, IL-6, IL-8, and IL-11). BCC growth is observed under the influence of the pro-inflammatory cytokine IL-6, which is induced by UVR in keratinocytes [90,91]. IL-6 activates angiogenesis in the human BCC cell line by elevating bFGF levels through both the PI3/Akt kinase and Janus kinase (JAK)/STAT3 pathways. Furthermore, IL-6 plays a crucial role in cancer progression by activating STAT3 and stimulating the proliferation and migration cancer cells. Jee et al. [92] presented that COX-2 blockage by siRNA reduces angiogenic activity in IL-6 overexpressing BCC cells. Angiogenic factors including GM-CSF, IL-8, CSF, VEGF, and monocyte chemotactic protein-1 (MCP-1) induced by IL-6 lead to cancer cell invasion in in vitro conditions. IL-6 is shown to have a key function in SCC progression by regulating a complex network of cytokines and proteases, and the tumor invasion induced by it is supported by MMP-1 overexpression [93].

The MMP family is mainly responsible for extracellular matrix (ECM) modification and degradation seen during invasive processes [94,95,96]. In normal skin conditions, MMPs are not constitutively expressed but can be secreted in response to exogenous factors such as UVR. As we have shown in our previous research, exposure to UVR leads to enhanced expression of MMP-1, -3, and -9 in irradiated skin [97]. Furthermore, MMP-2 and -9 have been linked with the metastatic and invasive potential of cancer cells [96,98,99]. MMPs not only contribute to cancer development by degrading the ECM but also by releasing sequestered growth factors, e.g., suppressing tumor cell apoptosis, b-fibroblast growth factor (b-FGF), VEGF, or TGF-β [96].

MMP-2 is an enzyme-degrading type IV collagenase that stimulates the secretion of active MMP-2 by TNF-α and activates processes involved in wound healing and cancer cell invasion. Type IV collagen and laminin contained in basement membrane components are potential substrates for MMP-2, so the activation of MMP-2 by TNF-α in human skin is particularly important in inducing angiogenesis and metastasis [100].

TNF-α may upregulate malignant melanoma invasion as well as migration in vitro [101]. Thus, TNF-α signaling is mainly studied in pro-inflammatory cytokines in skin carcinogenesis. It was discovered that not only TNF-α receptor subtypes but also transcription factors of the AP-1 family as well as protein kinase C-alpha (PKC-α) are involved in the cancer promotion-mediated inflammation, proliferation, invasion, and angiogenesis of tumors [14,68,102].

The pro-inflammatory action of TNF-α plays an important role in the early stages of carcinogenesis. It was revealed that TNF-α-deficient mice were resistant to the development of benign and malignant skin cancers triggered by repeated exposure to dimethylbenz(a)anthracene (DMBA). The observed resistance was connected to a markedly reduced inflammatory response in the dermis of mice. Interestingly, in the subsequent steps of carcinogenesis, TNF-α had no effect since the induced cancer in both TNF-α-deficient and wild-type animals presented comparable rates of malignancy progression. TNF-α initiates the activation of NF-κB signaling, leading to the initiation of various antiapoptotic factors [102,103]. TNF-α and TGFβ1 upregulate cyclooxygenase-1 (COX-1) and COX-2 expressions of mast cells and PG generation, which substantially affects skin carcinogenesis development by enhancing epidermal proliferation and stimulating inflammation within a cancer [104]. Increased TGF-β is usually connected with poor prognosis [63].

COX-2 is usually expressed in tumor cells, exerting a multifaceted role in promotion of carcinogenesis and inducing cancer cell resistance to radio- and chemotherapy. The upregulation of COX-2 expression is frequently caused by chronic exposure to UVR. Moreover, it is observed that most skin cancers overexpress COX-2. COX-2 is an enzyme that increases the production of PG, including PGE2, which is associated with tumor invasion, progression, and metastasis. Increased levels of PGE2 are especially noticed in BCC and SCC. Moreover, PGE2 may correlate with an enhanced propensity for invasive and metastatic behavior [105]. It was proved in a mouse model that COX-2-overexpressing transgenic animals [106] are highly vulnerable to skin cancer development, while COX-2 knockout mice are less susceptible to induced oncogenesis [107].

The IL-17/IL-23 axis of inflammation plays a crucial role in the development of skin cancers [71,108,109]. IL-23 and IL-12 belong to the family of heterodimeric cytokines that shares the 40 kD subunit. Interestingly, the deletion of IL-23a results in a marked reduction in DMBA/TPA-induced skin carcinogenesis, while the deletion of IL-12 results in a marked increase in skin tumorigenesis. Animals with a deletion of the common p40 subunit, which is shared by both IL-12 and IL-23, also did not develop skin cancer [110]. IL-23 is crucial for IL-17 expression.

IL-17 and IL-22 produced by Th17 cells enhance skin cancer promotion by activating STAT3 in stromal cells and tumors and by increasing the infiltration of myeloid cells in skin cancer environments [111,112,113]. Moreover, IL-11 and IL-6 drive the malignant development of tumor cells by the activation of STAT3 as well as by the upregulation of angiogenic and inflammatory factors [99,114].

## 6. Anti-Tumor Immune Molecules

Anti-tumor immune molecules IL-12 as well as interferon-gamma (IFN-γ) play crucial roles in preventing the development of skin cancers. IL-12 is a heterodimeric cytokine consisting of two subunits: p40 and p35. The p40 subunit is shared with IL-23, which is believed to be a cancer-promoting molecule. Mice with IL-12 ablation (knocked out p35 subunit) showed rapid tumor growth, suggesting that IL-12 has an anti-tumor function [110].

Similarly, in mice models, skin cancer presented a major role for IFN-γ in anti-tumor immunity [92,115]. It was observed that mice lacking an IFN-γ receptor or its downstream signaling STAT1 mediator were susceptible to the chemical carcinogen induction of sarcoma [116,117].

Immunomodulatory cytokines play a dual but sometimes controversial role in the process of carcinogenesis and inflammation. TGF-β and IL 10 produced mainly by T lymphocytes (Treg), dendritic cells (DC), macrophages, epithelial cells, and other immune and stromal cells belong to major immune-modulating cytokines in skin cancer.

The skin under the influence of UV radiation produces and activates Treg, which inhibits Th1-induced immunity against skin cancer through the production of IL-10 [118,119]. In addition, IL-10 knockout animals have been shown to be resistant to UVR-induced skin carcinogenesis, demonstrating the pro-tumor role of IL-10. However, the adoptive transfer of UVR-induced regulatory T cells from IL-10-deficient mice failed to suppress Th1 responses to skin cancer [120], suggesting that IL-10 merely limits anti-tumor adaptive immunity during skin cancer development.

TGF-β signaling was suggested to be critical for an early wave of Treg generation [120,121]. TGF-β also promotes the differentiation of naive T lymphocytes into peripheral Treg cells, which causes the effect of prolonged inflammation in animals and humans [122]. However, the role of TGF-β in the process of carcinogenesis is not so clear. This cytokine reduces both inflammation, which prevents neoplasms, and reduces the body’s anti-neoplastic resistance; hence, the final resultant effect of its action depends on the individual’s immune response in the cancer microenvironment [123]. Since TGF-β inhibits keratinocyte proliferation, its inactivation causes a rapid increase in keratinocyte count and skin thickening in a mouse model [124]. Moreover, papilloma development was enhanced in mice lacking TGF-β signaling and progressed to SCC with a predisposition to metastasis and angiogenesis [125]. Furthermore, the skin-specific ablation of TGF-β receptor 2 (TGF-β R-2) resulted in enhanced skin carcinogenesis induced by K-Ras activation or action of DMBA [126]. Therefore, on the one hand, TGF-β inhibits the development of primary skin tumors by limiting the inflammatory pathways that promote cancer development and by acting directly on transformed epithelial cells. However, once a tumor develops, TGF-β promotes the metastasis of many cancers, including skin malignances [127,128], by activating the epithelial–mesenchymal transition (EMT) process and by increasing cell mobility and tissue invasion [128,129]. Therefore, depending on the stage of tumor development, blocking TGF-β signaling and other immune modulating cells and cytokines in skin cancer may or may not be beneficial and circumstance-specific.

## 7. Conclusions

There is evidence suggesting that chronic inflammation is one of the major hallmarks of solar UVR and agent-mediated skin cancers. HIF-1α, STAT3, NF-κB, and their gene products, such as cytokines, COX-2, chemokines, and chemokine receptors, play a critical role in skin inflammation and carcinogenesis. Understanding the pathways of carcinogenesis seems crucial to understanding the potential role of inflammation in the initiation, promotion, and progression of skin cancer and its metastasis. Inflammatory pathways could be attractive targets for skin cancer prevention. Considering the potential role of inflammation in carcinogenesis, the reduced exposure to known skin carcinogens, and the ongoing studies evaluating the role of various pro-inflammatory mediators in carcinogenesis, their assessment as potential targets for the chemoprevention of skin cancers needs to be enhanced.

## Figures and Tables

**Table 1:** Inflammation molecules involved in different processes of carcinogenesis.

Stage:	Initiation	Promotion	Angiogenesis	Progression	Metastasis	Antitumor
IL-1		X				
IL-6	X	X	X	X		
IL-8		X	X			
IL-10	X			X		
IL-11		X				
IL-12						X
IL-17		X				
IL-23		X				
TGF-β	X			X		
TNF-α	X	X	X	X	X	
IFN-γ						X
HIF-1α	X					
GM-CSF			X			
CSF VEGF			X			

## Data Availability

No new data were created or analyzed in this study. Data sharing is not applicable to this article.

## References

[1] Boda D., Docea A.O., Calina D., Ilie M.A., Constantin C., Zurac S., Neagu M., Constantin C., Branisteanu D.E., Voiculescu V. (2018). Human papilloma virus: Apprehending the link with carcinogenesis and unveiling new research avenues (Review). Int. J. Oncol..

[2] Medzhitov R. (2008). Origin and physiological roles of inflammation. Nature.

[3] Calleja-Agius J., Brincat M., Borg M. (2013). Skin connective tissue and ageing. Best Pract. Res. Clin. Obstet. Gynaecol..

[4] Fisher G.J., Kang S., Varani J., Bata-Csorgo Z., Wan Y., Datta S., Voorhees J.J. (2002). Mechanisms of Photoaging and Chronological Skin Aging. Arch. Dermatol..

[5] Schauena M., Hornig-Doa H., Schomberga S., Herrmann G., Wiesner R.J. (2007). Mitochondrial electron transport chain activity is not involved in ultraviolet A (UVA)-induced cell death. Free Radic. Biol. Med..

[6] Penga Y., Xuanb M., Leunga V.Y.L., Cheng B. (2015). Stem cells and aberrant signaling of molecular systems in skin aging. Ageing Res. Rev..

[7] Minutoli L., Puzzolo D., Rinaldi M., Irrera N., Marini H.R., Arcoraci V., Bitto A., Crea G., Pisani A., Squadrito F. (2016). ROS-Mediated NLRP3 Inflammasome Activation in Brain, Heart, Kidney, and Testis Ischemia/Reperfusion Injury. Oxidative Med. Cell. Longev..

[8] Petersen A.B., Gniadecki R., Vicanova J., Thorn T., Wulf H.C. (2000). Hydrogen peroxide is responsible for UVA-induced DNA damage measured by alkaline comet assay in HaCaT keratinocytes. J. Photochem. Photobiol. B Biol..

[9] Sollberger G., Strittmatter G.E., Grossi S., Garstkiewicz M., Auf dem Keller U., French L.E., Beer H.D. (2015). Caspase 1 activity is required for UVB induced apoptosis of human keratinocytes. J. Investig. Dermatol..

[10] Ortiz M.L., Kumar V., Martner A., Mony S., Donthireddy L., Condamine T., Seykora J., Knight S.C., Malietzis G., Lee G.H. (2015). Immature myeloid cells directly contribute to skin tumor development by recruitin. J. Exp. Med..

[11] Grivennikov S.I., Greten F.R., Karin M. (2010). Immunity, Inflammation, and Cancer. Cell.

[12] Coussens L.M., Werb Z. (2002). Inflammation and cancer. Nature.

[13] Landskron G., De la Fuente M., Thuwajit P., Thuwajit C., Hermoso M.A. (2014). Chronic inflammation and cytokines in the tumor microenvironment. J. Immunol. Res..

[14] Mantovani A., Allavena P., Sica A., Balkwill F. (2008). Cancer related inflammation. Nature.

[15] Neagu M., Constantin C., Dumitrascu G., Lupu A., Caruntu C., Boda D., Zurac S. (2015). Inflammation markers in cutaneous melanoma edgy biomarkers for prognosis. Discoveries.

[16] Kricker A., Armstrong B.K., English D.R., Heenan P.J. (1995). Does intermittent sun exposure cause basal cell carcinoma? A case-control study in Western Australia. Int. J. Cancer.

[17] Zak-Prelich M., Narbutt J., Sysa-Jedrzejowska A. (2004). Environmental risk factors predisposing to the development of basal cell carcinoma. Dermatol. Surg..

[18] Mercuri S.R., Brianti P., Dattola A., Bennardo L., Silvestri M., Schipani G., Nisticò S.P. (2018). CO2 laser and photodynamic therapy: Study of efficacy in periocular BCC. Dermatol. Ther..

[19] Dattola A., Silvestri M., Bennardo L., Passante M., Scali E., Patruno C., Nisticò S.P. (2020). Role of Vitamins in Skin Health: A Systematic Review. Curr. Nutr. Rep..

[20] Ciążyńska M., Bednarski I.A., Narbutt J., Lesiak A. (2020). NLRP1 and NLRP3 inflammasomes as a new approach to skin carcinogenesis (Review). Oncol. Lett..

[21] Diffey B.L. (2002). Sources and measurement of ultraviolet radiation. Methods.

[22] Sample A., Zhao B., Qiang L., He Y.-Y. (2017). Adaptor protein p62 promotes skin tumor growth and metastasis and is induced by UVA radiation. J. Biol. Chem..

[23] Balupillai A., Nagarajan R.P., Ramasamy K., Govindasamy K., Muthusamy G. (2018). Caffeic acid prevents UVB radiation induced photocarcinogenesis through regulation of PTEN signaling in human dermal fibroblasts and mouse skin. Toxicol. Appl. Pharmacol..

[24] Marteijn J.A., Lans H., Vermeulen W., Hoeijmakers J.H.J. (2014). Understanding nucleotide excision repair and its roles in cancer and ageing. Nat. Rev. Mol. Cell Biol..

[25] Pfeifer G.P., Besaratinia A. (2012). UV wavelength-dependent DNA damage and human non-melanoma and melanoma skin cancer. Photochem. Photobiol. Sci..

[26] Gallagher R.P., Lee T.K. (2006). Adverse effects of ultraviolet radiation: A brief review. Prog. Biophys. Mol. Biol..

[27] Ji C., Yang B., Yang Z., Tu Y., Yang Y.-L., He L., Bi Z.-G. (2012). Ultra-violet B (UVB)-induced skin cell death occurs through a cyclophilin D intrinsic signaling pathway. Biochem. Biophys. Res. Commun..

[28] Svobodova A., Zdarilova A., Mališková J., Mikulková H., Walterova D., Vostálová J. (2007). Attenuation of UVA-induced damage to human keratinocytes by silymarin. J. Dermatol. Sci..

[29] Drobetsky E.A., Turcotte J., Chateauneuf A. (1995). A role for ultraviolet A in solar mutagenesis. Proc. Natl. Acad. Sci. USA.

[30] Gruijl D., Dijk V., Loveren V. (1999). UVB exposure-induced systemic modulation of Th1-and Th2-mediated immune responses. Immunology.

[31] Ebisz M., Brokowska M. (2015). Harmful impact of ultraviolet radiation on human skin. Hygeia Public Health.

[32] Borkowska B., Kardynał A., Słowińska M., Maj M., Sicińska J., Czuwara J., Piekarczyk E., Szymańska E., Kurzeja M., Warszawik-Hendzel O. (2013). Czerniak u osób korzystających z urządzeń opalających emitujących promienie UV (solariów). Prz. Dermatol..

[33] Ichihashi M., Ueda M., Budiyanto A., Bito T., Oka M., Fukunaga M., Tsuru K., Horikawa T. (2003). UV-induced skin damage. Toxicology.

[34] Ruetze M., Dunckelmann K., Schade A., Reuschlein K., Mielke H., Weise J.M., Gallinat S., Wenck H., Knott A. (2011). Damage at the root of cell renewal—UV sensitivity of human epidermal stem cells. J. Dermatol. Sci..

[35] Herrling T., Jung K., Fuchs J. (2008). The role of melanin as protector against free radicals in skin and its role as free radical indicator in hair. Spectrochim. Acta Part A Mol. Biomol. Spectrosc..

[36] Divya S.P., Wang X., Pratheeshkumar P., Son Y.O., Roy R.V., Kim D., Dai J., Hitron J.A., Wang L., Asha P. (2015). Blackberry extract inhibits UVB-induced oxidative damage and inflammation through MAP kinases and NF-κB signaling pathways in SKH-1 mice skin. Toxicol. Appl. Pharmacol..

[37] Salucci S., Burattini S., Curzi D., Buontempo F., Martelli A.M., Zappia G., Falcieri E., Battistelli M. (2014). Antioxidants in the prevention of UVB-induced keratynocyte apoptosis. J. Photochem. Photobiol. B Biol..

[38] Baier J., Maisch T., Maier M., Landthaler M., Bäumler W. (2007). Direct detection of singlet oxygen generated by UVA irradiation in human cells and skin. J. Investig. Dermatol..

[39] Filip A., Daicoviciu D., Clichici S., Bolfa P., Catoi C., Baldea I., Bolojan L., Olteanu D., Muresan A., Postescu I. (2011). The effects of grape seeds polyphenols on SKH-1 mice skin irradiated with multiple doses of UV-B. J. Photochem. Photobiol. B Biol..

[40] Choi K.-S., Kundu J.K., Chun K.-S., Na H.-K., Surh Y.-J. (2014). Rutin inhibits UVB radiation-induced expression of COX-2 and iNOS in hairless mouse skin: p38 MAP kinase and JNK as potential targets. Arch. Biochem. Biophys..

[41] Swalwell H., Latimer J., Haywood R.M., Birch-Machin M.A. (2012). Investigating the role of melanin in UVA/UVB- and hydrogen peroxide-induced cellular and mitochondrial ROS production and mitochondrial DNA damage in human melanoma cells. Free Radic. Biol. Med..

[42] Furue M., Tsuji G., Mitoma C., Nakahara T., Chiba T., Morino-Koga S., Uchi H. (2015). Gene regulation of filaggrin and other skin barrier proteins via aryl hydrocarbon receptor. J. Dermatol. Sci..

[43] Sutter C.H., Yin H., Li Y., Mammen J.S., Bodreddigari S., Stevens G., Cole J.A., Sutter T.R. (2009). EGF receptor signaling blocks aryl hydrocarbon receptor-mediated transcription and cell differentiation in human epidermal keratinocytes. Proc. Natl. Acad. Sci. USA.

[44] Fritsche E., Schafer C., Calles C., Bernsmann T., Bernshausen T., Wurm M., Hubenthal U., Cline J.E., Hajimiragha H., Schroeder P. (2007). Lightening up the UV response by identification of the arylhydrocarbon receptor as a cytoplasmatic target for ultraviolet B radiation. Proc. Natl. Acad. Sci. USA.

[45] Pollet M., Shaik S., Mescher M., Frauenstein K., Tigges J., Braun S.A., Sondenheimer K., Kaveh M., Bruhs A., Meller S. (2018). The AHR represses nucleotide excision repair and apoptosis and contributes to UV-induced skin carcinogenesis. Cell Death Differ..

[46] Vogeley C., Esser C., Tuting T., Krutmann J., Haarmann-Stemmann T. (2019). Role of the Aryl Hydrocarbon Receptor in Environmentally Induced Skin Aging and Skin Carcinogenesis. Int. J. Mol. Sci..

[47] Frauenstein K., Sydlik U., Tigges J., Majora M., Wiek C., Hanenberg H., Abel J., Esser C., Fritsche E., Krutmann J. (2013). Evidence for a novel anti-apoptotic pathway in human keratinocytes involving the aryl hydrocarbon receptor, E2F1, and checkpoint kinase 1. Cell Death Differ..

[48] Lin W.W., Karin M. (2007). A cytokine-mediated link between innate immunity, inflammation, and cancer. J. Clin. Investig..

[49] Neagu M., Constantin C., Manda G., Margaritescu I. (2009). Biomarkers of metastatic melanoma. Biomark. Med..

[50] Matti M., Lovászi M., Garzorz N., Atenhan A., Quaranta M., Lauffer F., Eyerich S. (2018). Sebocytes contribute to skin inflammation by promoting the differentiation of T helper 17 cells. Br. J. Dermatol..

[51] Nagy I., Pivarcsi A., Kis K., Koreck A., Bodai L., McDowell A., Seltmann H., Patrick S., Zouboulis C.C., Kemény L. (2006). Propionibacterium acnes and lipopolysaccharide induce the expression of antimicrobial peptides and proinflammatory cytokines/chemokines in human sebocytes. Microbes Infect..

[52] Alestas T., Ganceviciene R., Fimmel S., Müller-Decker K., Zouboulis C.C. (2005). Enzymes involved in the biosynthesis of leukotriene B4 and prostaglandin E2 are active in sebaceous glands. J. Mol. Med..

[53] Egeblad M., Nakasone E.S., Werb Z. (2010). Tumors as Organs: Complex Tissues that Interface with the Entire Organism. Dev. Cell.

[54] Qing-Yuan Z., Bing L., Yin-Ting C., Yin-Ping H., Feng W.-P., Wu Y., Long G.-H., Zou Y.N., Liu Y., Lin B.-Q. (2021). Gender differences in UV-induced skin inflammation, skin carcinogenesis and systemic damage. Environ. Toxicol. Pharmacol..

[55] Thornton M.J. (2002). The biological actions of estrogens on skin. Exp. Dermatol..

[56] Harnish D.C., Scicchitano M.S., Adelman S.J., Lyttle C.R., Karathanasis S.K. (2000). The role of CBP in estrogen receptor cross-talk with nuclear factor-kappaB in HepG2 cells. Endocrinology.

[57] Speir E., Yu Z.X., Takeda K., Ferrans V.J., Cannon R.O. (2000). Competition for p300 regulates transcription by estrogen receptors and nuclear factor-kappaB in human coronary smooth muscle cells. Circ. Res..

[58] Kim J., Modlin R.L., Moy R.L., Dubinett S.M., McHugh T., Nickoloff B.J., Uyemura K. (1995). IL-10 production in cutaneous basal and squamous cell carcinomas. A mechanism for evading the local T cell immune response. J. Immunol..

[59] Katiyar S.K. (2007). UV-induced immune suppression and photocarcinogenesis: Chemoprevention by dietary botanical agents. Cancer Lett..

[60] Vink A.A., Moodycliffe A.M., Shreedhar V., Ullrich S.E., Roza L., Yarosh D.B., Kripke M.L. (1997). The inhibition of antigen-presenting activity of dendritic cells resulting from UV irradiation of murine skin is restored by in vitro photorepair of cyclobutane pyrimidine dimers. Proc. Natl. Acad. Sci. USA.

[61] Meeran S.M., Mantena S.K., Katiyar S.K. (2006). Prevention of Ultraviolet Radiation–Induced Immunosuppression by (−)-Epigallocatechin-3-Gallate in Mice Is Mediated through Interleukin 12–Dependent DNA Repair. Clin. Cancer Res..

[62] Schwarz A., Stander S., Berneburg M., Bohm M., Kulms D., van Steeg H., Grosse-Heitmeyer K., Krutmann J., Schwarz T. (2002). Interleukin-12 suppresses ultraviolet radiation-induced apoptosis by inducing DNA repair. Nat. Cell Biol..

[63] Fan Y., Mao R., Yang J. (2013). NF-κB and STAT3 signaling pathways collaboratively link inflammation to cancer. Protein Cell.

[64] Parkin D.M. (2006). The global health burden of infection-associated cancers in the year 2002. Int. J. Cancer.

[65] Lu H., Ouyang W., Huang C. (2006). Inflammation, a Key Event in Cancer Development. Mol. Cancer Res..

[66] Hanahan D., Weinberg R.A. (2000). The Hallmarks of Cancer. Cell.

[67] Staudt L.M. (2010). Oncogenic activation of NF-kappaB. Cold Spring Harb Perspect Biol..

[68] Balkwill F.R., Coussens L.M. (2004). An inflammatory link. Nat. Cell Biol..

[69] Karin M., Greten F.R. (2005). NF-κB: Linking inflammation and immunity to cancer development and progression. Nat. Rev. Immunol..

[70] Cho M.L., Kang J.W., Moon Y.M., Nam H.J., Jhun J.Y., Heo S.B., Jin H.-T., Min S.-Y., Ju J.-H., Park K.-S. (2006). STAT3 and NF-kappaB signal path¬way is required for IL-23-mediated IL-17 production in spontaneous arthritis animal model IL-1 recep¬tor antagonist-deficient mice. J. Immunol..

[71] Oppmann B., Lesley R., Blom B., Timans J.C., Xu Y., Hunte B., Vega F., Yu N., Wang J., Singh K. (2000). Novel p19 Protein Engages IL-12p40 to Form a Cytokine, IL-23, with Biological Activities Similar as Well as Distinct from IL-12. Immunity.

[72] Singh A., Singh A., Bauer S.J., Wheeler D.L., Havighurst T.C., Kim K., Verma A.K. (2016). Genetic deletion of TNFα inhibits ultraviolet radiation-induced development of cutaneous squamous cell carcinomas in PKCε transgenic mice via inhibition of cell survival signals. Carcinogenesis.

[73] Michael N.L., Moore J.P. (1999). HIV-1 entry inhibitors: Evading the issue. Nat. Med..

[74] Shao Y., Le K., Cheng H., Aplin A.E. (2015). NF-κB Regulation of c-FLIP Promotes TNFα-Mediated RAF Inhibitor Resistance in Melanoma. J. Investig. Dermatol..

[75] Karin M. (2006). NF-κB and cancer: Mechanisms and targets. Mol. Carcinog..

[76] Greten F.R., Eckmann L., Greten T.F., Park J.M., Li Z.-W., Egan L.J., Kagnoff M.F., Karin M. (2004). IKKβ Links Inflammation and Tumorigenesis in a Mouse Model of Colitis-Associated Cancer. Cell.

[77] Bell S., Degitz K., Quirling M., Jilg N., Page S., Brand K. (2003). Involvement of NF-kappaB signalling in skin physiology and disease. Cell Signal..

[78] Naugler W.E., Karin M. (2008). NF-κB and cancer—identifying targets and mechanisms. Curr. Opin. Genet. Dev..

[79] Prasad S., Ravindran J., Aggarwal B.B. (2009). NF-κB and cancer: How intimate is this relationship. Mol. Cell. Biochem..

[80] Zhu Z., Zhong S., Shen Z. (2011). Targeting the inflammatory pathways to enhance chemotherapy of cancer. Cancer Biol. Ther..

[81] Yu H., Kortylewski M., Pardoll D. (2007). Crosstalk between cancer and immune cells: Role of STAT3 in the tumour microenvironment. Nat. Rev. Immunol..

[82] Hanahan D., Weinberg R.A. (2011). Hallmarks of Cancer: The Next Generation. Cell.

[83] Fridman W.H., Pagès F., Sautès-Fridman C., Galon J. (2012). The immune contexture in human tumours: Impact on clinical outcome. Nat. Rev. Cancer.

[84] Grivennikov S.I., Karin M. (2010). Dangerous liaisons: STAT3 and NF-kappaB collaboration and cross-talk in cancer. Cytokine Growth Factor Rev..

[85] Schreiber R.D., Old L.J., Smyth M.J. (2011). Cancer Immunoediting: Integrating Immunity’s Roles in Cancer Suppression and Promotion. Science.

[86] Medler T.R., Coussens L.M. (2014). Duality of the immune response in cancer: Lessons learned from skin. J. Investig. Dermatol..

[87] Dunn G.P., Bruce A.T., Ikeda H., Old L.J., Schreiber R.D. (2002). Cancer immunoediting: From immunosurveillance to tumor escape. Nat. Immunol..

[88] Shanmugam M.K., Nguyen A.H., Kumar A.P., Tan B.K., Sethi G. (2012). Targeted inhibition of tumor proliferation, survival, and metastasis by pentacyclic triterpenoids: Potential role in prevention and therapy of cancer. Cancer Lett..

[89] Cataisson C., Salcedo R., Hakim S., Moffitt B.A., Wright L., Yi M., Stephens R., Dai R.M., Lyakh L., Schenten D. (2012). IL-1R-MyD88 signaling in keratinocyte transformation and carcinogenesis. J. Exp. Med..

[90] Schwarz T., Luger T.A. (1989). Effect of UV irradiation on epidermal cell cytokine production. J. Photochem. Photobiol. B.

[91] Chung J.H., Youn S.H., Koh W.S., Eun H.C., Cho K.H., Park K.C., Youn J.I. (1996). Ultraviolet B Irradiation-Enhanced Interleukin (IL)-6 Production and mRNA Expression Are Mediated by IL-1α in Cultured Human Keratinocytes. J. Investig. Dermatol..

[92] Jee S.-H., Chu C.-Y., Chiu H.-C., Huang Y.-L., Tsai W.-L., Liao Y.-H., Kuo M.-L. (2004). Interleukin-6 Induced Basic Fibroblast Growth Factor-Dependent Angiogenesis in Basal Cell Carcinoma Cell Line via JAK/STAT3 and PI3-Kinase/Akt Pathways. J. Investig. Dermatol..

[93] Lederle W., Depner S., Schnur S., Obermueller E., Catone N., Just A., Fusenig N.E., Mueller M.M. (2010). IL-6 promotes malignant growth of skin SCCs by regulating a network of autocrine and paracrine cytokines. Int. J. Cancer.

[94] Hua H., Li M., Luo T., Yin Y., Jiang Y. (2011). Matrix metalloproteinases in tumorigenesis: An evolving paradigm. Cell. Mol. Life Sci..

[95] Pytliak M., Vargová V., Mechírová V. (2012). Matrix Metalloproteinases and Their Role in Oncogenesis: A Review. Onkology.

[96] Ravi R., Piva T.J., Vereecken D.P. (2013). The role of furin in the development of skin cancer. Highlights in Skin Cancer.

[97] Ciążyńska M., Bednarski I.A., Wódz K., Narbutt J., Sobjanek M., Woźniacka A., Lesiak A. (2018). Impact of Ultraviolet Radiation on Expression of Transforming Growth Factor β, Smad2, Metalloproteinases-1, -3, -8, -9, Cathepsin K and Progerin. Photochem. Photobiol..

[98] Ramos M.C., Steinbrenner H., Stuhlmann D., Sies H., Brenneisen P. (2004). Induction of MMP-10 and MMP-1 in a squamous cell carcinoma cell line by ultraviolet radiation. Biol. Chem..

[99] Dong K.K., Damaghi N., Picart S.D., Markova N.G., Obayashi K., Okano Y., Masaki H., Grether-Beck S., Krutmann J., Smiles K.A. (2008). UV-induced DNA damage initiates release of MMP-1 in human skin. Exp. Dermatol..

[100] Han Y.-P., Tuan T.-L., Wu H., Hughes M., Garner W.L. (2001). TNF-alpha stimulates activation of pro-MMP2 in human skin through NF-(kappa)B mediated induction of MT1-MMP. J. Cell Sci..

[101] Katerinaki E., Evans G.S., Lorigan P.C., MacNeil S. (2003). TNF-α increases human melanoma cell invasion and migration in vitro: The role of proteolytic enzymes. Br. J. Cancer.

[102] Mueller M.M. (2006). Inflammation in epithelial skin tumours: Old stories and new ideas. Eur. J. Cancer.

[103] Scott K.A., Arnott C.H., Robinson S.C., Moore R.J., Thompson R.G., Marshall J.F., Balkwill F.R. (2004). TNF-α regulates epithelial expression of MMP-9 and integrin αvβ6 during tumour promotion. A role for TNF-α in keratinocyte migration?. Oncogene.

[104] Pérez-Lorenzo R., Markell L.M., Hogan K.A., Yuspa S.H., Glick A.B. (2010). Transforming growth factor β1 enhances tumor promotion in mouse skin carcinogenesis. Carcinogenesis.

[105] Singh T., Katiyar S.K. (2011). Green tea catechins reduce invasive potential of human melanoma cells by targeting COX-2, PGE2 receptors and epithelial-to-mesenchymal transition. PLoS ONE.

[106] Müller-Decker K., Neufang G., Berger I., Neumann M., Marks F., Fürstenberger G. (2002). Transgenic cyclooxygenase-2 overexpression sensitizes mouse skin for carcinogenesis. Proc. Natl. Acad. Sci. USA.

[107] Tiano H.F., Loftin C.D., Akunda J., Lee C.A., Spalding J., Sessoms A., Dunson D.B., Rogan E.G., Morham S.G., Smart R.C. (2002). Deficiency of either cyclooxygenase (COX)-1 or COX-2 alters epidermal differentiation and reduces mouse skin tumorigenesis. Cancer Res..

[108] Trinchieri G., Pflanz S., Kastelein R.A. (2003). The IL-12 family of heterodimeric cytokines: New players in the regulation of T cell responses. Immunity.

[109] Vignali D.A., Kuchroo V.K. (2012). IL-12 family cytokines: Immunological playmakers. Nat. Immunol..

[110] Langowski J.L., Zhang X., Wu L., Mattson J.D., Chen T., Smith K., Basham B., McClanahan T., Kastelein R.A., Oft M. (2006). IL-23 promotes tumour incidence and growth. Nat. Cell Biol..

[111] Wang L., Yi T., Zhang W., Pardoll D.M., Yu H. (2010). IL-17 Enhances Tumor Development in Carcinogen-Induced Skin Cancer. Cancer Res..

[112] Forcales S.V., Albini S., Giordani L., Malecova B., Cignolo L., Chernov A., Coutinho P., Saccone V., Consalvi S., Williams R. (2011). Signal-dependent incorporation of MyoD-BAF60c into Brg1-based SWI/SNF chromatin-remodelling complex. EMBO J..

[113] Nardinocchi L., Sonego G., Passarelli F., Avitabile S., Scarponi C., Failla C.M., Simoni S., Albanesi C., Cavani A. (2015). Interleukin-17 and interleukin-22 promote tumor progression in human nonmelanoma skin cancer. Eur. J. Immunol..

[114] Gu D., Fan Q., Zhang X., Xie J. (2012). A Role for Transcription Factor STAT3 Signaling in Oncogene Smoothened-driven Carcinogenesis*. J. Biol. Chem..

[115] Ikeda H., Old L.J., Schreiber R.D. (2002). The roles of IFN gamma in protection against tumor development and cancer immunoediting. Cytokine Growth Factor Rev..

[116] Kaplan D.H., Shankaran V., Dighe A.S., Stockert E., Aguet M., Old L.J., Schreiber R.D. (1998). Demonstration of an interferon -dependent tumor surveillance system in immunocompetent mice. Proc. Natl. Acad. Sci. USA.

[117] Street S.E.A., Cretney E., Smyth M.J. (2001). Perforin and interferon-γ activities independently control tumor initiation, growth, and metastasis. Blood.

[118] Chaudhry A., Samstein R.M., Treuting P., Liang Y., Pils M.C., Heinrich J.-M., Jack R.S., Wunderlich F.T., Brüning J.C., Müller W. (2011). Interleukin-10 Signaling in Regulatory T Cells Is Required for Suppression of Th17 Cell-Mediated Inflammation. Immunity.

[119] Loser K., Apelt J., Voskort M., Mohaupt M., Balkow S., Schwarz T., Grabbe S., Beissert S. (2007). IL-10 controls ultraviolet-induced carcinogenesis in mice. J. Immunol..

[120] Josefowicz S.Z., Lu L.-F., Rudensky A.Y. (2012). Regulatory T Cells: Mechanisms of Differentiation and Function. Annu. Rev. Immunol..

[121] Vignali D.A.A., Collison L.W., Workman C.J. (2008). How regulatory T cells work. Nat. Rev. Immunol..

[122] Bettelli E., Carrier Y., Gao W., Korn T., Strom T.B., Oukka M., Weiner H.L., Kuchroo V.K. (2006). Reciprocal developmental pathways for the generation of pathogenic effector TH17 and regulatory T cells. Nature.

[123] Glick A.B. (2012). The Role of TGFβSignaling in Squamous Cell Cancer: Lessons from Mouse Models. J. Ski. Cancer.

[124] Wang X.J., Greenhalgh D.A., Bickenbach J.R., Jiang A., Bundman D.S., Krieg T., Derynck R., Roop D.R. (1997). Expression of a dominant-negative type II transforming growth factor beta (TGF-beta) receptor in the epidermis of transgenic mice blocks TGF-beta-mediated growth inhibition. Proc. Natl. Acad. Sci. USA.

[125] Go C., Li P., Wang X.J. (1999). Blocking transforming growth factor beta signaling in transgenic epidermis accelerates chemical carcinogenesis: A mechanism associated with increased angiogenesis. Cancer Res..

[126] Lu S.-L., Herrington H., Reh D., Weber S., Bornstein S., Wang D., Li A.G., Tang C.-F., Siddiqui Y., Nord J. (2006). Loss of transforming growth factor-beta type II receptor promotes metastatic head-and-neck squamous cell carcinoma. Genes Dev..

[127] Derynck R., Akhurst R.J., Balmain A. (2001). TGF-β signaling in tumor suppression and cancer progression. Nat. Genet..

[128] Katsuno Y., Lamouille S., Derynck R. (2013). TGF-beta signaling and epithelial-mesenchymal transition in cancer progression. Curr. Opin. Oncol..

[129] Kalluri R., Weinberg R.A. (2009). The basics of epithelial-mesenchymal transition. J. Clin. Investig..

